# Medical Law and Medical School Curricula: A Systematic Review

**DOI:** 10.7759/cureus.54377

**Published:** 2024-02-17

**Authors:** Eylon Arbel, Alyssa Reese, Kenny Oh, Archana Mishra

**Affiliations:** 1 School of Medicine, University at Buffalo Jacobs School of Medicine and Biomedical Sciences, Buffalo, USA; 2 Department of Surgery, Lewis Katz School of Medicine, Philadelphia, USA; 3 Internal Medicine, University at Buffalo Jacobs School of Medicine and Biomedical Sciences, Buffalo, USA

**Keywords:** ethics, medical humanities, medical student, medical law, health law, medical education

## Abstract

Health law plays a crucial role in the field of medicine, as it dictates appropriate practices, regulations, and rights and responsibilities for healthcare professionals and patients. Despite this undeniable relationship, there is a lack of focus on health law, and an outdated hidden curriculum in medical education has perpetuated long-standing negative perceptions of the legal system.

PubMed was searched for articles related to medicolegal education that were published from January 1950 to December 2022. The following search terms were utilized: “(medical student) AND (law OR legal OR medico-legal) AND (education)”. Literature that directly or indirectly discussed the relationship between law and medicine as well as the role of medical student education within the medicolegal nexus were reviewed. Additional literature was identified from reference lists of systematic and literature reviews. The authors manually reviewed each included publication to determine key details, study populations, and conclusions.

The PubMed search revealed 3,592 papers that were sorted for relevance. Forty-four articles published between 1971 and 2022 were reviewed and analyzed. Three main themes consistently emerged from the discussions in these articles. The first theme concerns the sentiment among medical students that they were ill-prepared to manage the legal aspects of healthcare. The second theme concerns the negative perception of health law by medical students. The third theme details the benefits of including medicolegal courses in medical school curricula.

This study sheds light on the notion that medical students feel ill-prepared to handle the legal aspects of healthcare due to limited medicolegal education. Furthermore, negative perceptions of the legal field continue to exist amongst medical students due to a plethora of factors, including an outdated hidden curriculum. Incorporating medicolegal courses into medical school curricula can foster positive attitudes toward the field of law and lead to enhanced professional ethics, increased patient advocacy, and potentially improved patient outcomes.

## Introduction and background

Medical law plays a crucial role in the field of medicine because it dictates appropriate practices, regulations, and rights and responsibilities for health care professionals and patients. Despite the undeniable connection between the medical and legal systems, there is a long-standing perception that the fields of medicine and law are at odds. Although there has been a notable shift in attitudes and perspectives in recent years, medical students commonly learn about health law from a hidden curriculum that tends to reinforce this outdated belief [1-6]. This hidden curriculum is composed of the implicit or unspoken messages, attitudes, and norms that shape student perception.

As the intricate relationship between medicine and law continues to evolve, the need to educate and prepare medical students for future encounters with legal medicine, including medical ethics, becomes increasingly important. In the United States, medical education is often divided into one to two years of didactic training and two to three years of clinical training. In contrast, internationally, the structure and duration of medical education differs and may span five to six years.

A comprehensive understanding of the interplay between law and medicine can help medical students advocate for health policy reform, identify medical practices that fail to meet standards of care, navigate ethical dilemmas, and inform patients of their rights and legal matters pertaining to the social determinants of health, such as housing issues, income maintenance, health care access, and social service care [7-9]. This systematic review highlights the importance of incorporating medical-legal education into medical school curricula.

## Review

Methods

**Figure 1:**
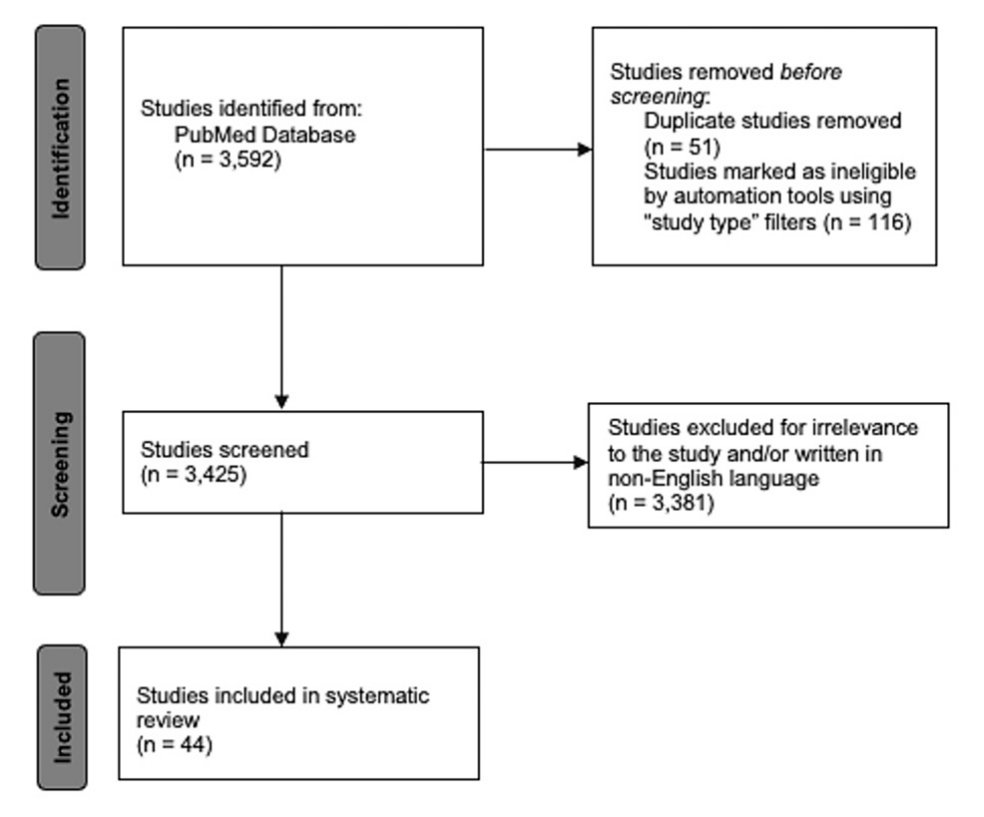
Literature selection PRISMA flowchart Literature selection visualization. PRISMA: Preferred Reporting Items for Systematic Reviews and Meta-Analyses

PubMed was searched for articles published from January 1952 to December 2022 related to medicolegal education. The following search terms were used: (medical student) AND (law OR legal OR medico-legal) AND (education). These terms yielded 3,592 publications, of which 51 duplicates and 116 papers of ineligible study types, such as clinical trials were excluded. The remaining 3,425 publications were then manually sorted by the authors. Of these, 3,381 were excluded for irrelevance or being written in a non-English language. Literature that discussed the relationship between law and medicine as well as the role of medical student education within the medicolegal nexus were reviewed. No limitation was placed on the inclusion of articles based on medical students' year of study, age or gender of students, or questionnaire pattern. This resulted in a final collection of 44 publications. A flowchart depicting the selection process for this review can be found in Figure 1. The Preferred Reporting Items for Systematic Reviews and Meta-Analyses (PRISMA) flowchart template was utilized in the creation of Figure 1.

Results 

A total of 3,592 papers were identified and sorted for relevance. Forty-three articles published between 1971 and 2022 in the United States, United Kingdom, Arab Gulf Cooperation Council, France, Australia, South Korea, Canada, Poland, and Germany were included and reviewed [1-44]. The authors then reviewed each included publication to determine key details, study populations, and conclusions. The data from the final pool of included publications were used to fill in Table 1 as an organized platform to compare the literature. Three main themes emerged from the articles. The first theme concerns the negative perception of health law by medical students. The second theme dictates the sentiment among medical students that they were ill-prepared to manage the legal aspects of health care. The third theme details the benefits of including medicolegal courses in medical school curricula. The reviewed literature is summarized in Table 1.

**Table 1:** Thematic categorization of reviewed publications ^a^Theme 1, medical students have negative perceptions of the legal field; Theme 2, medical students feel ill-prepared to handle legal aspects of health care; Theme 3, incorporation of medicolegal courses in medical school curricula benefits students.

Theme(s)^a^	Citation	Location	Aim	Study Category
1	Johnston et al. 2014 [4]	California, USA	Characterize exposure to defensive medicine during medical school rotations	Survey
1	Kelly and Miller 2009 [3]	United States	Evaluate medical student perceptions of the U.S. medical malpractice system	Survey
2	Anderson et al. 2020 [1]	California, USA	Explore medical student understanding of informed consent	Survey
2	Annandale et al. 1996 [5]	United Kingdom	Understand medical students' knowledge of malpractice, attitudes toward litigation, and the significance of litigation in their future practice	Survey
3	Al-Azri NH 2020 [10]	Arab Gulf Cooperation Council	Address the need for a medical law curriculum in medical schools	Review
3	Andrews MD 1991 [11]	Oklahoma City, OK, USA	Describe an approach to teaching about ethical and legal issues	Course Description
3	Campbell AT 2012 [12]	United States/United Kingdom	Recommendations for teaching law in medical schools	Review/ Editorial
3	Dowie A 2014 [14]	United Kingdom	Assessment techniques for medical ethics and law	Commentary
3	DuVal MK 1971 [6]	United States	Emphasize the need to improve medical student understanding of the legal profession	Editorial/ Commentary
3	Fenwick A 2014 [16]	United Kingdom	Guide teachers on what, when, and how to assess medical students' learning of medical ethics and law	Commentary
3	Fitchett et al. 2011 [17]	United Kingdom	Highlight the benefit of integration of human rights education into medical ethics and law curricula in medical school	Commentary
3	Gard et al. 2021 [19]	United States	Scoping literature review to identify best practices in medical-legal partnerships and training methods	Literature Review
3	Girard et al. 2020 [20]	United States	Discuss a pilot study with a legislative advocacy track for preclinical medical students	Survey
3	Jenkins and Lemak 2007 [22]	Gainesville, FL, USA	Describe a semester-long mock trial program	Case Study
3	Kapp et al. 2012 [23]	United States	Examine legal risk management and ethical patient care information denoted by medical school faculty	Survey
3	Kapp MB 2018 [8]	Tallahassee, FL, USA	Describe an individualized medicine-law elective	Course Description
3	Koehler and McMenamin 2012 [25]	Australia	Examine the adequacy of a medical law tutorial program	Survey
3	Lawson CM 1987 [26]	United States	Discuss interdisciplinary medical law seminar	Course Description
3	Lee et al. 2020 [28]	South Korea	Explore the creation of an interdisciplinary course involving law	Course Description/ Survey
3	Liu et al. 2005 [29]	Canada	Evaluate the impact of interdisciplinary medical ethics and legal issues sessions on medical students	Survey
3	Magwood et al. 2003 [30]	Canada	Describe the medical humanities program	Program Description
3	Marcinkowski JT 2009 [31]	Poland	Discuss methods of interprofessional education between medical and law students	Commentary
3	Margetts JK 2016 [32]	United Kingdom	Proposals for developing medical education in law	Literature Review
3	Mills et al. 2012 [33]	United States	Outline a curriculum on health policy for medical students	Curriculum/ Commentary
3	Naitove BJ 1982 [34]	United States	Discuss problems inherent in current interdisciplinary education and outline a program for medical and law students	Review/ Editorial
3	O’Neill et al. 1990 [35]	Santa Rosa, CA, USA	Describe a course on medical ethics, jurisprudence, and economics	Course Description
3	Olick RS 2001 [7]	Iowa City, IA, USA	Discuss a biomedical ethics and health law program	Course Description
3	Peabody et al. 2008 [36]	United States	Describe an interdisciplinary medicolegal issues seminar series	Course Description/ Survey
3	Pettignano et al. 2017 [37]	United States	Describe a medical-legal practice curriculum	Course Description/ Survey
3	Quraishi et al. 2005 [40]	United States	Describe a medical school’s Health Policy and Legislative Awareness initiative	Course Description/ Survey
3	Rosseau et al. 2022 [41]	United States	Discuss the importance of criminal justice education for medical students	Editorial
3	Schildmann et al. 2018 [42]	Germany	Evaluate a course on risks and errors in medical sciences with ethical and legal aspects	Course Description/ Survey
3	Walters et al. 1973 [9]	Australia	Describe an interdisciplinary exercise for medical and law students	Course Description/ Survey
3	Wlasienko P 2005 [43]	Poland	Emphasize the need for ethical and legal education in medical student education	Commentary
1, 3	LeBlang et al. 1985 [27]	United States	Assess the impact of the required curriculum in medical law	Survey/ Commentary
1, 3	O'Leary et al. 2012 [2]	United States	Assess medical student and resident experiences with defensive medicine	Survey
1, 3	Preston-Shoot and McKimm 2010 [39]	United Kingdom	Determine how UK medical are teaching law and ethics	Survey
2, 3	Cohen et al. 2010 [13]	United States	Describe how medical-legal partnerships teach residents, medical students, and other providers to address socially caused health disparities	Program Evaluation
2, 3	Felthous and Miller 1987 [15]	United States	Determine what medical law curriculum is being taught at U.S. medical schools	Survey
2, 3	Franchitto and Rouge 2010 [18]	France	Determine how medical students appraise the teaching of legal medicine curriculum	Survey
2, 3	Grumet BR 1979 [21]	United States	Survey of medical student interest and the state of medical-legal curriculum in the United States	Survey
2, 3	Knight and Thompson 1986 [24]	United Kingdom	Evaluate the teaching of legal medicine and ethics	Course Description/Survey
2, 3	Faihs et al. 2022 [44]	Austria	Evaluate teaching of ethics, law, and decision-making skills	Survey
1, 2, 3	Preston-Shoot et al. 2011 [38]	United Kingdom	Assess the evidence base for teaching law in medical school	Survey

Discussion

Theme 1: Medical Students Have Negative Perceptions of the Legal Field

Historically, medical students and resident physicians have harbored negative attitudes and perceptions toward the legal field. O’Leary et al. found that few experienced healthcare professionals try to alter negative perceptions among medical students and many even encourage avoidance rather than interdisciplinary collaboration during their education [2]. Additionally, the authors noted that a significant portion of surveyed medical students and residents reported being explicitly taught by attending physicians to consider liability when making clinical decisions [2]. Thus, while healthcare professionals typically take malpractice liability into consideration when making clinical decisions, the guidance of medical students to embrace defensive medical practices is not always the appropriate solution and can perpetuate the poor relations between the fields of medicine and law. A survey conducted at Brown Medical School in 2006 by Kelly and Miller revealed drastically negative perceptions of the legal field and the medical malpractice system, especially in students intending to specialize in high-risk areas [3]. These negative perceptions included the notion that the current United States medical malpractice system is functioning poorly, needs major changes, and includes many lawsuits that are brought when no physician negligence or incompetence has occurred. These perceptions were not significantly different between students’ first and fourth years, further emphasizing the need for curricula that portray the realm of legal medicine as a tool rather than an obstacle.

Theme 2: Medical Students Feel Ill-Prepared to Handle the Legal Aspects of Healthcare

The notion that medical students feel ill-prepared to handle the legal aspects of health care is not surprising considering that many medical schools in the United States do not require medicolegal education beyond lectures addressing ethical dilemmas and medical malpractice. In 1979, medical schools that included medicolegal education in their curriculum required approximately 13 hours of instruction [21]. Although discussion of ethics is now required in medical curricula, the limited medicolegal education in medical school can translate to a lack of fundamental clinical skills. These skills include discussing informed consent, collaborating with medical-legal partnerships, understanding confidentiality, and applying relevant laws in the clinical setting regarding child abuse, abortion, and involuntary hospitalization [1,13,27].

Although a medicolegal curriculum is important, it must be accompanied by proper assessment in order to evaluate student proficiency and ensure future application of knowledge. As of 2023, only Texas, Maine, and Kansas require separate medical jurisprudence examinations for physicians to become licensed in the state [15]. Although it may not be necessary for physicians to pass a separate examination for licensing purposes, an adequate understanding and assessments at the medical school level, such as including medicolegal assessment in board examinations, are needed to thoroughly prepare students.

Theme 3: Incorporation of Medicolegal Courses in Medical School Curricula Benefits Students

Despite the prevalence of negative perceptions, we must acknowledge that a sound understanding of the law is essential for resolving medical issues and being an ethical physician. Thankfully, there is evidence that medicolegal education can improve the perceptions medical students have of the field of law. Preston-Shoot et al. reported increased positive perceptions of law among medical students after formal legal teaching had taken place [38]. Additionally, LeBlang et al. found that medical students’ attitudes toward the law improved after they completed a specialized educational program [27]. Comparisons of pretest and posttest responses revealed that attitudes toward the legal system, interprofessional cooperation, and medical malpractice crises improved favorably after medicolegal instruction. Thus, these studies demonstrate that medical curricula have the potential to address preformed biases from the start of medical school. 

The noted benefits of including medicolegal coursework range from lessening liability risks to improving interdisciplinary collaboration and professional ethics. Additionally, medicolegal education enables physicians to advocate for patients, lobby for policy changes, and bridge the gap between legislation and clinical medicine in a way that improves patient outcomes [12].

Strategies to improve medical students' understanding of legal medicine include individualized medicine-law electives, mock trials, and simulations [7-9,13,22,28,30,33,35-37,40,42]. If possible, collaborations between local law and medical school faculty can optimize the use of institutional resources while providing students the opportunity to interact and mitigate negative perceptions and biases. Medicolegal education can be incorporated in each phase of medical school: preclinical years of training can focus on understanding medical law, and clinical years of training can focus on exploring and applying this knowledge. Information surrounding medicolegal topics can be incorporated into ongoing patient-care courses and clerkships by identifying potential legal barriers to care during a standardized patient encounter or by discussing the use of medical-legal partnerships during a workshop on social determinants of health. 

## Conclusions

Preparing future physicians to navigate complex legal discussions, recognize and address legal issues, advocate for patient rights, manage risks, and contribute to policy is crucial. Thus, the authors encourage the creative expansion of medical school curricula to include simple yet impactful approaches to teaching and assessing medical students' knowledge in this area throughout all years of medical school. In doing so, we will ensure that physicians are prepared to foster a positive relationship with the field of law and improve interdisciplinary collaboration and patient outcomes.
